# Editorial: Editor’s Pick 2021: Highlights in Cell Growth and Division

**DOI:** 10.3389/fcell.2022.859568

**Published:** 2022-03-07

**Authors:** Philipp Kaldis

**Affiliations:** Department of Clinical Sciences, Lund University, Clinical Research Centre (CRC), Malmö, Sweden

**Keywords:** cell biology, developmental biology, cell growth, cell division, metabolism, neural development

The section “Cell Growth and Division” of Frontiers in Cell and Developmental Biology was started 6 years ago with the aim to capture exciting research in the broadest possible term of cell and developmental biology. This section has come a long way and after publishing approximately 900 manuscript, it is well established.

For the year 2021, we decided to launch a “research topic” named “*Editor’s Pick 2021: Highlights in Cell Growth and Division*” to highlight some of the exciting manuscript that were published. The goal was to capture and highlight some of the exciting manuscripts, which reflect this section’s goals in the broadest sense. Therefore, you should not be surprised to find topics like neural (stem cell) differentiation (Ly and Wang; Yan et al.), spermatogenesis (Rahman and Pang; Zheng et al.), hair development (Fukuda et al.), neural development (Su et al.), genetic mouse engineering (Jang et al.), hearing loss (Zhong et al.; Yang et al.), cell cycle (Creff and Besson; Jang et al.; Ly and Wang), cytokinins (Gibb et al.), gastric cancer (Jiang et al.), cardiomyocyte regeneration (Bo et al.), and metabolism (Su et al.; Bo et al.). Although all these topics are exciting and are well reflected in our section, we seek manuscripts also in other emerging research fields that are connected to cell and developmental biology.

I would like to thank all the authors of the manuscripts of the research topic “*Editor’s Pick 2021: Highlights in Cell Growth and Division*” for their contribution to science, to their research field, and to our section. At the same time, I should highlight the efforts of all the associate editors and review editors without whom we could not run a successful journal.

Since we have now started a new year, I am looking forward to a lot of exciting research and hope that some of it will be published in our section.

